# Genome-Wide Analysis of the AP2/ERF Transcription Factors Family and the Expression Patterns of *DREB* Genes in Moso Bamboo (*Phyllostachys edulis*)

**DOI:** 10.1371/journal.pone.0126657

**Published:** 2015-05-18

**Authors:** Huili Wu, Hao Lv, Long Li, Jun Liu, Shaohua Mu, Xueping Li, Jian Gao

**Affiliations:** 1 International Center for Bamboo and Rattan, Key Laboratory of Bamboo and Rattan Science and Technology, State Forestry Administration, Beijing, People’s Republic of China; 2 Hunan Forest Botanical Garden, Changsha, Hunan Province, People’s Republic of China; University of Missouri, UNITED STATES

## Abstract

The AP2/ERF transcription factor family, one of the largest families unique to plants, performs a significant role in terms of regulation of growth and development, and responses to biotic and abiotic stresses. Moso bamboo (*Phyllostachys edulis*) is a fast-growing non-timber forest species with the highest ecological, economic and social values of all bamboos in Asia. The draft genome of moso bamboo and the available genomes of other plants provide great opportunities to research global information on the AP2/ERF family in moso bamboo. In total, 116 AP2/ERF transcription factors were identified in moso bamboo. The phylogeny analyses indicated that the 116 AP2/ERF genes could be divided into three subfamilies: AP2, RAV and ERF; and the ERF subfamily genes were divided into 11 groups. The gene structures, exons/introns and conserved motifs of the PeAP2/ERF genes were analyzed. Analysis of the evolutionary patterns and divergence showed the PeAP2/ERF genes underwent a large-scale event around 15 million years ago (MYA) and the division time of AP2/ERF family genes between rice and moso bamboo was 15–23 MYA. We surveyed the putative promoter regions of the *PeDREBs *and showed that largely stress-related cis-elements existed in these genes. Further analysis of expression patterns of *PeDREBs* revealed that the most were strongly induced by drought, low-temperature and/or high salinity stresses in roots and, in contrast, most *PeDREB* genes had negative functions in leaves under the same respective stresses. In this study there were two main interesting points: there were fewer members of the PeDREB subfamily in moso bamboo than in other plants and there were differences in *DREB* gene expression profiles between leaves and roots triggered in response to abiotic stress. The information produced from this study may be valuable in overcoming challenges in cultivating moso bamboo.

## Introduction

Plants are exposed to many types of environmental conditions during their life cycle. The extremes of the major environmental conditions, namely soil moisture, salt concentration and temperature, limit the growth, development, productivity and geographical distribution of plants across the globe and reduce potential values [1, 2]. When a plant is subjected to abiotic stresses, a series of genes with diverse functions are induced or repressed. These proteins can be categorized into two groups: functional and regulatory proteins [3]. Transcription factors are an important group of regulatory proteins. The AP2/ERF (APETALA2/ethylene response factor) transcription factors are one of the most important groups in plants and are characterized by one or two AP2/ERF domains that consist of 60–70 conserved amino-acid residues, which perform a very important role in the plant’s stress defense mechanism [4–7]. The 147 AtAP2/ERF family genes and the 164 OsAP2/ERF family genes were divided into three subfamilies by Nakano et al. [7]: AP2, RAV and ERF. There are two soloist genes, At4g13040 and Os02g29550, in the AP2/ERF family in Arabidopsis and rice, respectively. The AP2 subfamily members, encoding two AP2/ERF domains [8, 9], have important functions in regulation of growth and development, including leaf epidermal cell identity [10], flower and ovule development [11, 12], spikelet meristem determinacy [13] and seed growth [14, 15]. The RAV subfamily transcription factors, possessing a single AP2/ERF domain and a B3-like domain [16–19], play significant roles in regulating expression of target genes in response to ethylene [20], brassinosteroids [21] and biotic and abiotic stresses [22, 23]. The ERF subfamily is further divided into two major subfamilies, the ERF and CBF/DREB subfamilies [5], containing a single conserved AP2/ERF domain. The ERF subfamily show specific functions in responding to biotic stress, for example, pathogen and disease stimuli [24]. However, the CBF/DREB subfamily transcription factors are involved in response to abiotic stress in plants, such as OsDREB1 for drought [25, 26], AtCBF1 for low-temperature [27], CaDREBLP1 for water deficit and high salinity [28], ZmDREB2A for heat [29], CkDREB for osmotic stress [30] and AtDREB1A for multiple stresses in transgenic plants, such as drought or cold stresses [31].

Bamboo is one of the most important non-timber forest products in the world [32]. About 2.5 billion people depend economically on bamboo, and international trade in bamboo amounts to over US$2.5 billion per year [33]. Moso bamboo, *Phyllostachys edulis* (Carrière) J. Houzeau (synonym *P*. *heterocycla* Carrière) is a large woody bamboo with the highest ecological, economic and cultural value of all bamboos in Asia, accounting for up to 70% of the total area of bamboo growth. Moso bamboo has been valued at US$5 billion of annual forest production in China [34, 35], used as timber, paper and art ware, and the shoots as delicious food. Disadvantageous climatic and environmental conditions limit the development and distribution of moso bamboo—such as drought or cold conditions in northern China, where moso bamboo grows poorly. Genome database research has produced remarkable results in terms of enhancing stress tolerance in plants, and the AP2/ERF family has been identified in Arabidopsis [5], rice [36,37], poplar [38], grapevine [9], wheat [39], peach [40], castor bean [41], cucumber [19], barley [42] and soybean [7]. The draft genome sequences of moso bamboo [32] and other plants provide a wonderful opportunity for a comparative genome survey of the AP2/ERF family transcription factors in moso bamboo.

In this study, the genes in the AP2/ERF family in the moso bamboo genome were surveyed. Phylogenetic, exon/intron, protein motif structure and evolutionary relationship analyses were performed on the AP2/ERF family in moso bamboo. Analyses of the putative promoter regions and expression patterns of the PeDREBs were also performed. The results will be useful in further study of the AP2/ERF family in plants.

## Materials and Methods

### Identification of AP2/ERF family genes in the moso bamboo genome

The conserved AP2/ERF domains of Arabidopsis AP2/ERF protein sequences were originally applied as seed sequences to search the National Center for Gene Research (http://www.ncgr.ac.cn/bamboo) [32] and the NCBI database (www.ncbi.nlm.nih.gov). The search was based on a BLASTP search with an expected value of 100. The identified AP2/ERF genes were used as queries to reconfirm the multiple databases to ensure that no additional related genes were missing from the database. All sequences that met the requirements were analyzed to eliminate genes that did not contain the known conserved domains and motifs using the Pfam database (http://pfam.janelia.org/) [43], the SMART database (http://smart.embl-heidelberg.de/) [44] and the Conserved Domain Database of the NCBI (http://www.ncbi.nlm.nih.gov/Structure/cdd/wrpsb.cgi) [45]. Bioinformatic analysis of AP2/ERF genes was performed using ExPASy (http://www.expasy.ch/tools/pi_tool.html) to determine the number of amino acids of the open reading frame (ORF), molecular weight (MW), isoelectric point (pI) and length of the open reading frame (length) for each gene. Subcellular localization was predicted using Softberry (http://linux1.softberry.com/). The AP2/ERF genes in Arabidopsis were searched using the Arabidopsis Information Resource TAIR (http://www.arabidopsis.org/), and the AP2/ERF genes in rice were obtained from the MSU database (http://rice.plantbiology.msu.edu/) and the Rice Genome Annotation Project (http://rice.plantbiology.msu.edu/index.shtml).

### Phylogenetic, motif recognition and gene structure analyses

The multiple alignment analysis was performed with ClustalX 1.83 software [46], and phylogenetic trees were generated by the neighbor-joining (NJ) method and bootstrap analysis (1000 replicates) [47] and displayed using MEGA 6.0 software [48]. The conserved motifs were analyzed using MEME version 4.9.1 [49, 50]. Gene structure was investigated using the Gene Structure Display Server websites (http://gsds.cbi.pku.edu.cn/).

### Evolutionary patterns and divergence of the AP2/ERF gene family in rice and moso bamboo analysis

Pairwise alignment of AP2/ERF gene encoding sequences of the orthologous and paralogous pairs was performed using ClustalX 1.83 software. The occurrence of duplication events and divergence of homologous genes, as well as selective pressure on duplicated genes, were estimated by calculating synonymous (Ks) and non-synonymous substitutions (Ka) per site between the duplicated gene-pairs using DnaSP version 5.10.1 [51, 52]. The Ks rate was considered as the proxy for time to estimate the dates of the gene-pair duplication events, and the dates of the duplication events were further deduced using the formula T = Ks/2λ, assuming that clock-like rates (λ) of rice and moso bamboo were 6.5 × 10^–9^ substitutions/synonymous site/year [32, 53, 54].

### Analysis of the putative promoter regions of the DREB gene subfamily in moso bamboo

The 2000-bp upstream sequences of the transcriptional start site of *PeDREB*s were chosen to identify the cis-elements in the putative promoter regions. The PLACE website (http://www.dna.affrc.go.jp/PLACE/) [55] was applied to identify the putative cis-regulatory elements along the promoter sequences.

### Plant treatment and qRT-PCR analysis

Moso bamboo seeds were collected from Guilin, Guang Xi Province, China. The seeds from individual plants were germinated on sterile filter papers in culture dishes, while keeping the filter papers moist and in darkness at 25°C. The seedlings were transferred to plastic pots containing vermiculite and grown in an illuminated incubator with 16/8 h of light/dark at 25/18°C and humidity of 80%, and watered with Hoagland nutrient solution every week. Plants were cultivated for three months.

For drought-stress treatments, a 20% PEG-6000 solution (and for salinity treatments, a 250 mM NaCl solution) was poured over the culture medium vermiculite. For low-temperature stress treatments, plants were transferred to an illuminated incubator at 4°C with other culture conditions unchanged. Plant leaves and roots were collected for analysis at 0, 0.5, 1, 3, 6, 12 and 24-h time points. Plant roots were rapidly washed with distilled water (4°C incubated water for low-temperature treatment) after treatment at each time-point, frozen immediately in liquid nitrogen and stored at—80°C prior to RNA extraction.

The total RNA was extracted using TRIZOL reagent (Invitrogen, Germany) based on the manufacturer’s instructions, and extensively pre-treated using RNase-free DNase I (Promega, Madison, WI, USA) to digest any genomic DNA. RNA quality was characterized initially on a 1% agarose gel with Tris–acetate–EDTA (TEA) buffer and NanoDrop 8000 spectrophotometer (Thermo Scientific) and then the integrity of RNA samples was further evaluated using an Agilent 2100 Bioanalyzer (USA). For first-strand cDNA synthesis, 2 μg of total RNA in a 20 μl reaction volume treated with DNase was transcribed using M-MLV reverse transcriptase in accordance with the manufacturer’s protocols (Promega).

Due to the small number of members characterized in the DREB subfamily, we analyzed the expression patterns of 24 DREB genes (excluding PH01000343G0780, PH01003107G0070 and PH01003928G0080, because of lack of specific primers) by using real-time quantitative PCR (qRT-PCR) reactions to survey their functions in response to abiotic stress. The qRT-PCR reactions were carried out with a LightCycler480H System (Roche) using SYBRH Premix EX TaqTM kit (Roche). The 20-μl reaction system contained 0.4 μl (10 mM) of each primer, 2 μl (20 ng) of cDNA, 10 μl of SYBR Green I Master and then ddH2O was added to make up the final volume, following the manufacturer’s instructions. Amplification reactions were performed as follows: 95°C for 10 s, 60°C for 10 s and 72°C for 20 s. All reactions were performed in triplicate, both technical and biological. The primers, listed in S1 Table, were chosen using Primer 3.0 software (http://www.genome.wi.mit.edu/cgi-bin/primer/primer3.cgi). Tonoplast intrinsic protein 41 gene (TIP41) [56] was used as the reference gene. The data of relative expression levels were calculated using the comparative ΔΔ^CT^ method, and presented in clusters using fold-change values transformed to log_2_ format and clustered by Multiple Array Viewer using the average method linkage method with Pearson’s correlation distance metric [57].

## Results

### Detection of AP2/ERF transcription factors

We identified a total of 121 putative AP2/ERF genes, predicted to include one or two complete or incomplete AP2/ERF domains. The ORF and gene lengths, MW, pI and subcellular localization of these putative genes were analyzed, and results are listed in S2 Table. Gene length ranged from 309 (PH01000069G0560) to 1926 bp (PH01001338G0140), MW from 11.15 (PH01000069G0560) to 64.71 kDa (PH01001338G0140) and pI from 4.45 (PH01002652G0240) to 11.07 (PH01002393G0230). Subcellular localization prediction indicated that 119 genes were located in the nucleus, with score ranging from 4.40 (PH01000084G1170) to 9.95 (PH01001938G0330); however, two genes (PH01003811G0100 and PH01000443G0450) may have been located in the chloroplast. Five of the 121 genes (PH01000303G0100, PH01000028G1600, PH01002652G0240, PH01004233G0070 and PH01195697G0010) were excluded from further analysis due to having a very small domain and resulting in an unacceptable phylogenetic tree. Therefore, the remaining 116 genes were used for phylogenetic analysis, which had corresponding locus IDs in the database National Center for Gene Research.

### Phylogenetic analysis of the AP2/ERF family

To determine the phylogenetic relationships between the genes in the moso bamboo AP2/ERF family, multiple alignment analyses were executed using amino acid sequences of the AP2/ERF domain. In total, 17 consensus residues, 4G, 12G, 18I, 31L, 32G, 34T, 41A, 42A, 44A, 45Y, 55D, 57A, 58A, 63G, 66A, 72N and 73F were more than 85% conserved among the 116 proteins in the PeAP2/ERF family (S1 Fig). The alignment indicated that 80 of these proteins were in the ERF subfamily, 28 were in the AP2 subfamily, seven were in the RAV subfamily and there was one soloist. Because 80 proteins possessed one AP2/ERF domain and shared high homology, they belonged to the ERF subfamily (S1 Fig). Three proteins, PH01000320G0460, PH01003811G0100 and PH01004434G0040, had C-terminal regions of the AP2/ERF domain that contained very low homology to the consensus sequence (S1 Fig)—these three proteins belonged to group VI–L. There were 17 proteins that included two AP2/ERF domains and these were in the AP2 subfamily; whereas, 11 proteins possessed one AP2/ERF domain that shared high homology with AP2 proteins, and so were classified in the AP2 subfamily (S1 Fig). Seven proteins each contained one AP2/ERF domain and one B3 domain and hence were classified into the RAV subfamily (S1 Fig).

**Fig 1:**
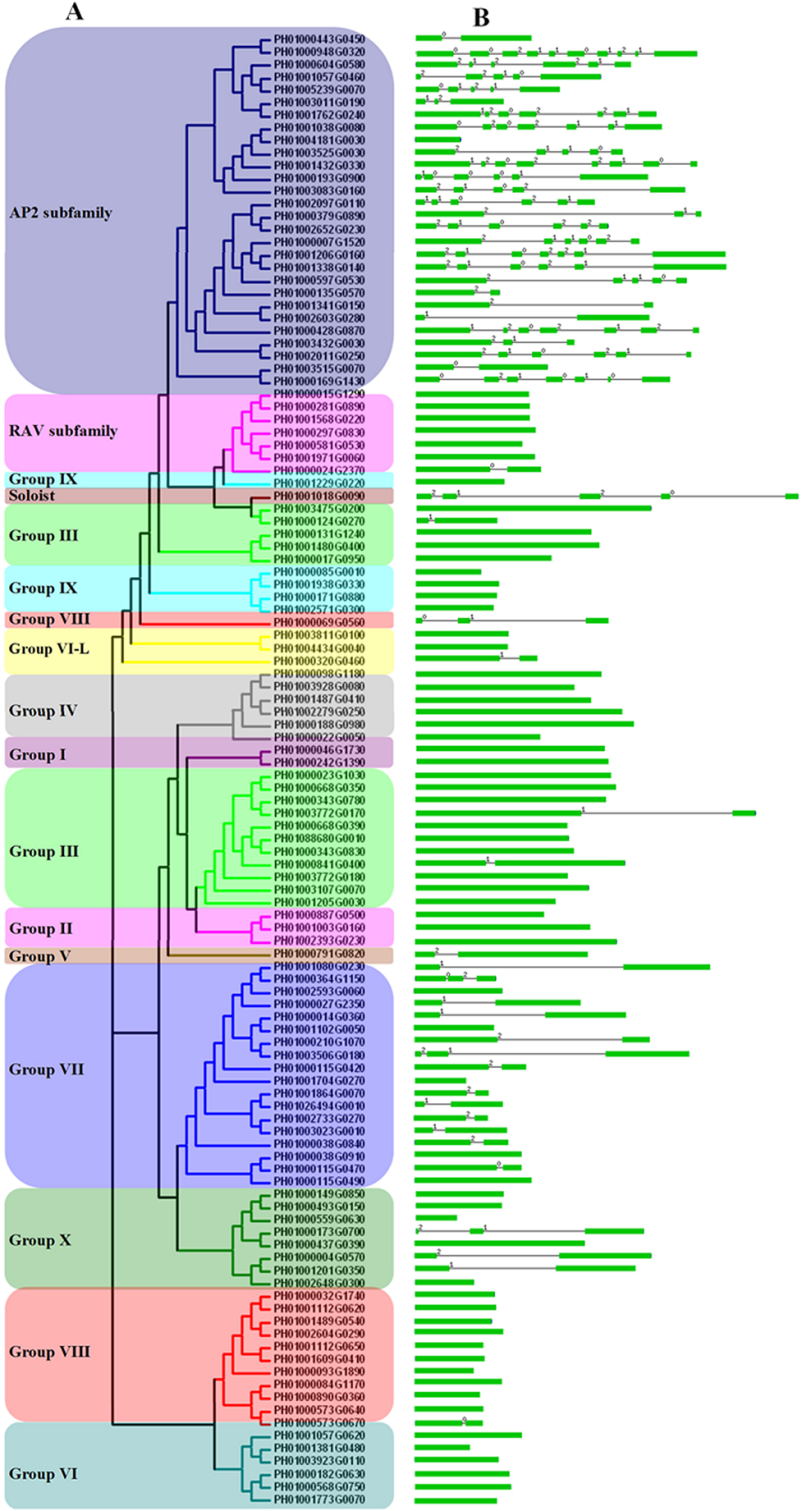
Unrooted phylogenetic tree and exons/intronsof moso bambooAP2/ERF family genes. (A) The phylogenetic tree was generatedusing the AP2/ERF domain amino acid sequences by the Clustal 1.83 software with the NJ method.Groups I–IV are CBF/DREB subfamily proteins, and groups V–X are ERF subfamily proteins. (B) The distribution of exons/introns within AP2/ERF genes. The green boxes represent exons, black lines linked two exons represent introns and the number over lines represents the splicing phases.

**Fig 2:**
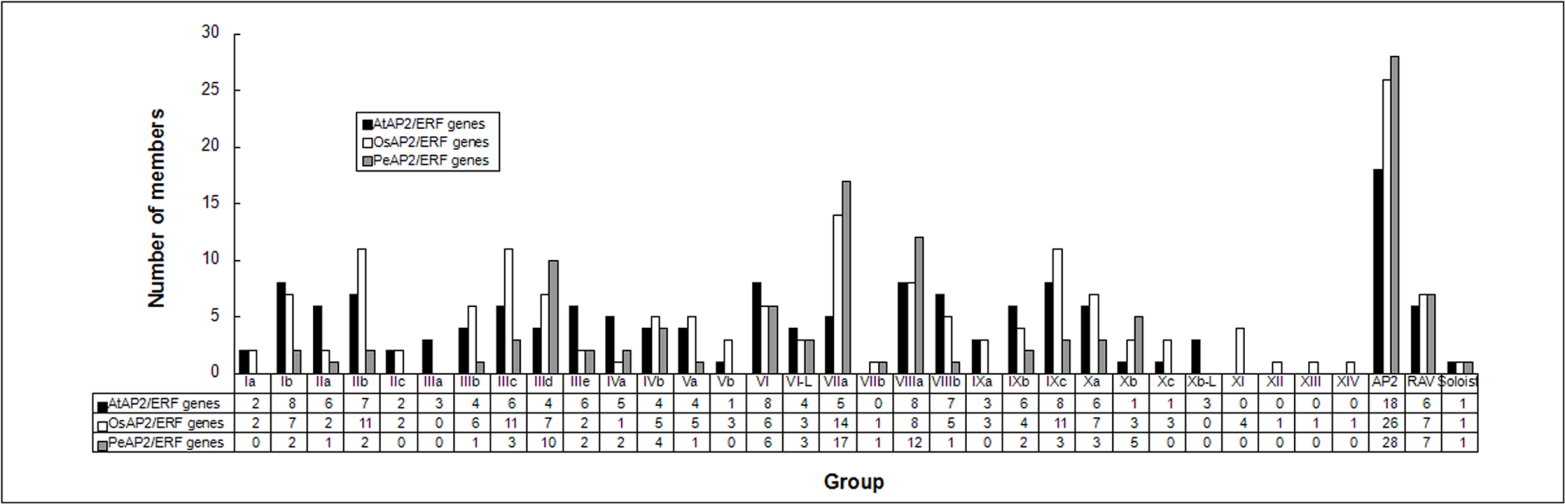
Comparison of group/subgroup size between moso bamboo, Arabidopsis and rice AP2/ERF families. The subclassification was by Nakano et al. (2006) [7].

Based on above observations, an unrooted phylogenetic tree with 116 PeAP2/ERF domain sequences was constructed (Fig 1A), and an additional tree was generated using the domain sequences of 135 AtAP2/ERF, 147 OsAP2/ERF and 116 PeAP2/ERF proteins (S2 Fig). These trees distinguished the AP2 subfamily; the RAV subfamily; groups I–VI, VI–L and VII–X; and a soloist. The reliability of the clustering was supported by the method described in Nakano et al. [7]. The numbers and members of groups I–X are shown in Fig 2 and Table 1. The classification of subgroups was analyzed by the presence and position of introns and the motifs within the AP2/ERF proteins, as described later. Several striking points were found in the PeAP2/ERF family: (1) no members belonged to groups Ia, IIc, Vb, IXa and Xc; (2) there were more members in groups IIId, VIIa, VIIIa and Xb than that from Arabidopsis and rice, whereas there were fewer in other groups; (3) group VIIb existed in moso bamboo and rice, but groups IIIa and Xb-L were specific to Arabidopsis; and (4) there were no members in groups XI—XIV, and these groups may be specific to rice (see Table 1 and Fig 2).

**Table 1:** The ERF subfamily genes in moso bamboo.

Group Name	ID Name	Gene Name
Ib	PH01000046G1730	PeDREB2A
Ib	PH01000242G1390	
IIa	PH01002393G0230	
IIb	PH01000887G0500	
IIb	PH01001003G0160	
IIIb	PH01001205G0030	
IIIc	PH01000017G0950	
IIIc	PH01000131G1240	
IIIc	PH01001480G0400	
IIId	PH01000023G1030	
IIId	PH01000668G0350	PeDREB1A
IIId	PH01000343G0780	
IIId	PH01003772G0170	
IIId	PH01000668G0390	
IIId	PH01088680G0010	
IIId	PH01000343G0830	
IIId	PH01000841G0400	
IIId	PH01003772G0180	
IIId	PH01003107G0070	
IIIe	PH01003475G0200	
IIIe	PH01000124G0270	
IVa	PH01000098G1180	PeDREB2
IVa	PH01003928G0080	PeDREB1
IVb	PH01001487G0410	
IVb	PH01002279G0250	
IVb	PH01000022G0050	
IVb	PH01000188G0980	
Va	PH01000791G0820	
VI	PH01001057G0620	
VI	PH01001381G0480	
VI	PH01003923G0110	
VI	PH01000182G0630	
VI	PH01000568G0750	
VI	PH01001773G0070	
VI-L	PH01000320G0460	
VI-L	PH01003811G0100	
VI-L	PH01004434G0040	
VIIa	PH01001080G0230	
VIIa	PH01000364G1150	
VIIa	PH01000027G2350	
VIIa	PH01000014G0360	
VIIa	PH01000210G1070	
VIIa	PH01003506G0180	
VIIa	PH01001102G0050	
VIIa	PH01000115G0420	
VIIa	PH01002733G0270	
VIIa	PH01003023G0010	
VIIa	PH01001864G0070	
VIIa	PH01026494G0010	
VIIa	PH01000038G0840	
VIIa	PH01000038G0910	
VIIa	PH01000115G0470	
VIIa	PH01000115G0490	
VIIa	PH01002593G0060	
VIIb	PH01001704G0270	
VIIIa	PH01000093G1890	
VIIIa	PH01000032G1740	
VIIIa	PH01001112G0620	
VIIIa	PH01000084G1170	
VIIIa	PH01000890G0360	
VIIIa	PH01000573G0640	
VIIIa	PH01000573G0670	
VIIIa	PH01001489G0540	
VIIIa	PH01002604G0290	
VIIIa	PH01001112G0650	
VIIIa	PH01001609G0410	
VIIIb	PH01000069G0560	
IXb	PH01000171G0880	
IXb	PH01002571G0300	
IXc	PH01001229G0220	
IXc	PH01000085G0010	
IXc	PH01001938G0330	
Xa	PH01000004G0570	
Xa	PH01001201G0350	
Xa	PH01002648G0300	
Xb	PH01001490G0850	
Xb	PH01000493G0150	
Xb	PH01000559G0630	
Xb	PH01000173G0700	
Xb	PH01000437G0390	

Note: The groups I to IV genes were DREB subfamily genes, the groups V, VI, VI-L, and VII to X genes were ERF subfamily genes.

### Gene structure and conserved motifs analysis of the AP2/ERF gene family

**Fig 3:**
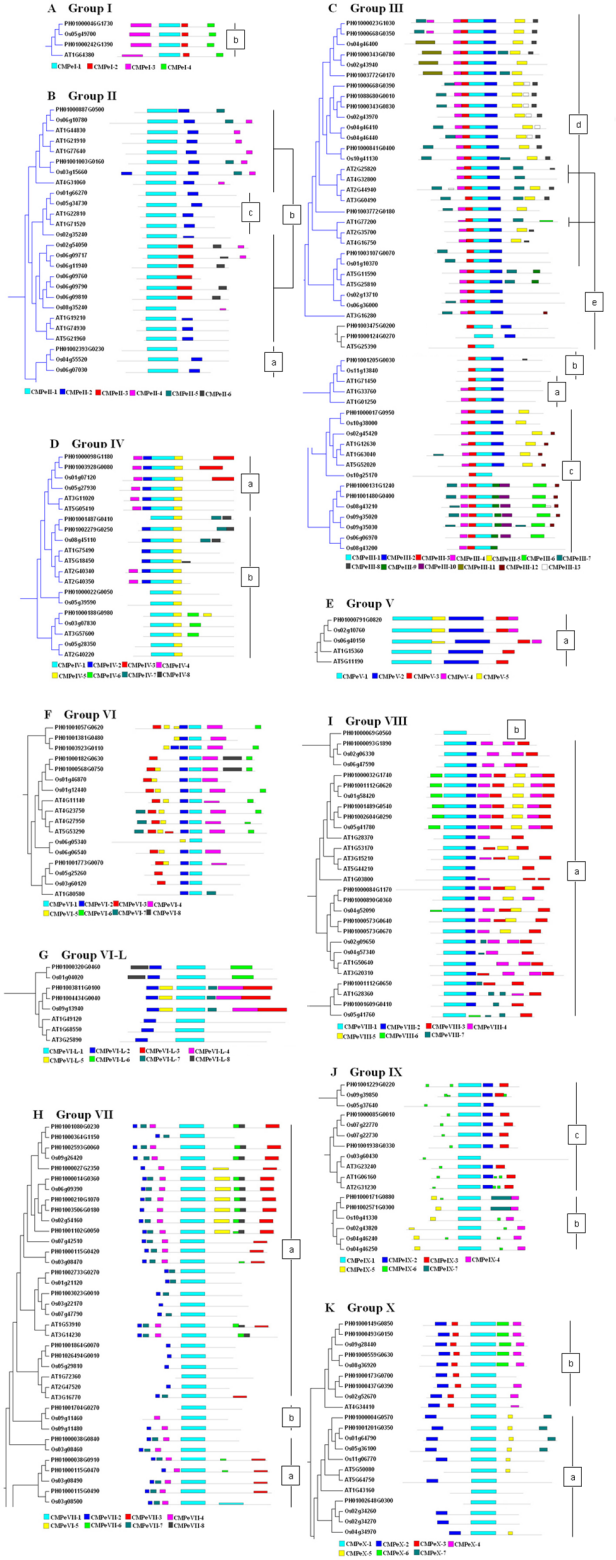
Gene motifs within each group of the ERF subfamily in moso bamboo, Arabidopsis and rice. The clades of the phylogenetic tree detected within moso bamboo, Arabidopsis and rice ERF subfamilies. The conserved motifs were identified in the proteins of every group respectively: groups I (A), II (B), III (C), IV(D), V (E), VI (F), VI–L (G), VII (H), VIII (I), IX (J) and X (K). Each colored box below the tree represents the conserved motifs. The CMPeI-1 to VI-1, VI-L-1, and VII-1 to X-1 represent the AP2/ERF domain in every group.

To further understanding of the structural diversity of moso bamboo AP2/ERF genes, we analyzed exon/intron organization within PeAP2/ERF family members. AP2 subfamily members had 1–9 introns (excluding PH01004181G0030), the soloist gene had four introns, and most members of group VII had one or two introns, whereas the other subfamily and groups seldom had introns (Fig 1B). To identify the potential motifs in the PeAP2/ERF family, full-length amino acid sequences of PeAP2/ERF genes and the homologous genes of Arabidopsis and rice were analyzed, and we listed the homologous clades with any PeAP2/ERF genes (see Fig 3 and S3 Fig). The motifs in different subfamilies or subgroups were different, including numbers and conserved sequences. For example, motif analysis identified CMPeII-4, CMPeIII-8, CMPeIII-12 and CMPeVII-3 motifs as LWSY motifs in groups IIb, IIId, IIIc and VIIa, respectively. The ERF-associated amphiphilic repression (EAR) motif of motifs CMPeIII-5 and CMPeVIII-3 in groups IIId, IIIc and VIIIa, was identified as a conserved sequence, LxLxLxLPP, in the C-terminal regions. CMPeVI-4, CMPeVII-6 and CMPeIX-4, identified as putative phosphorylation site sequences, were conserved in groups VI, VIIa and IXb. A unique motif, CMPeX-3, containing a characteristic consensus sequence, Cx_2_Cx_2_Cx_2~4_C, was conserved in the N-terminal region of members in group Xb. CMPeA-6 existed in PH01001341G0150, PH01002603G0280, Os12g03290 and Os11g03540 of AP2 subfamily proteins; and CMPeR-8 only existed in the C-terminal region of PH01000015G1290, PH01000281G0890 and PH00101568G0220 of RAV subfamily proteins. Of soloists (S3 Fig), PH01001018G0090 and Os02g29550 genes had higher homology than AT4G13040 (S3 Fig and S3 Table).

### Characteristics of each group in the PeAP2/ERF family

The characteristics of each group in the PeAP2/ERF family in association with AtAP2/ERF and OsAP2/ERF families were reported by Nakano et al. [7]. The AP2 and RAV subfamilies were characterized and the 11 groups within the ERF subfamily were further analyzed as follows. Group I only contained two genes in subgroup Ib, which had high homology with Os05g49700 and AT1G64380 genes in subgroup Ib; and there were no subgroup Ia genes in moso bamboo (Fig 3A). Group II was divided into two subgroups, IIa and IIb, and lacked subgroup IIc (Fig 3B). The motif analysis identified CMPeII-4 as a LWSY motif in the C-terminal region—similar to CMII-3 in subgroup IIb as described by Nakano et al. [7]. Os04g55520 and Os06g07030 genes in subgroup IIa and PH01002393G0230 gene were clustered into one clade, indicating that PH01002393G0230 belonged in subgroup IIa. Group III consisted of four subgroups, IIIb, IIIc, IIId and IIIe, with no subgroup IIIa (Fig 3C). The putative subgroup IIIc proteins, PH01000131G1240 and PH01001480G0400, contained three consensus motifs, CMPeIII-4, -9 and -12. The CMPeIII-4 and -9 motifs were similar to the CMIII-3 motif’s region I and region II on both sides of the AP2/ERF domain [7]; the CMPeIII-12 motif has also been identified as a LWSY motif. Despite the PH01000017G0950 protein containing motifs different to the former two proteins, these three proteins had no introns and were grouped into a single clade (Fig 1), so the PH01000017G0950 protein was also assigned to subgroup IIIc. The CMPeIII-5 (EAR motif) and -8 motifs were similar to CMIII-6 and CMIII-7, which were contained in subgroup IIId—so proteins containing CMPeIII-5 and -8 motifs were assigned to subgroup IIId and, further based on phylogeny, 10 proteins were assigned to subgroup IIId. PH01003475G0200 and PH01000124G0270 were grouped into a single clade and shared the same motifs, and so were assigned to subgroup IIIe—using a similar method, PH01001205G0030 was assigned to subgroup IIIb. Group IV was divided into two subgroups, IVa and IVb (Fig 3D). The CMPeIV-2 and -4 motifs were identified as similar to CMIV-1 and -2, which were conserved throughout the N-terminal region outside the AP2/ERF domain in subgroup IVa of Arabidopsis and rice; while PH01000098G1180 and PH01003928G0080 possessed the CMPeIV-2 and -4 motifs, and so were assigned to subgroup IVa. The phylogenetic tree and the other motif analysis indicated that the remaining proteins were from subgroup IVb. Followed the classification method of Nakano et al. [7], groups I–IV belonged to the CBF/DREB subfamily as described by Sakuma et al. [5], which was also consistent with the present study. In total, 27 proteins belonged to the CBF/DREB subfamily.

The ERF subfamily genes were divided into two subfamilies: CBF/DREB and ERF [7]. The genes of groups V, VI, VI–L and VII–X were ERF subfamily genes. Group V contained only one protein (Fig 3E), PH01000791G0820, sharing two motifs, CMPeV-2 and CMPeV-3, identified as similar to CMV-1 and CMV-2 which have been described in subgroup Va [7], so the PH01000791G0820 gene was assigned to subgroup Va; there were no subgroup Vb genes in moso bamboo. Group VI consisted of proteins that shared two conserved motifs, CMPeVI-3 and -5, in the N-terminal region (Fig 3F), and analysis indicated that they were similar to CMVI-1 and CMVI-2 [7]; the six genes in group VI had no introns (Fig 1B). PH01000320G0460, PH01003811G0100 and PH01004434G0040 shared a motif, CMPeVI-L-2, in the N-terminal region outside the AP2/ERF domain, similar to CMPeVI-3, and so these three genes were assigned to subgroups VI–L (Fig 3G). Group VII was divided into two subgroups: VIIa and VIIb (Fig 3H). There were 17 proteins that contained four motifs, CMPeVII-2, -3 and -4 were identified as similar to CMVII-1, -5 and -6, respectively, and were in subgroup VIIa of OsERF genes. Among the 17 proteins, 14 had one or two introns (Fig 1B), a phenomenon also described in Arabidopsis and rice [7]. Based on these analyses, the 17 proteins were assigned to subgroup VIIa. PH01001704G0270 had high homology to Os09g11460 protein, and so was assigned to subgroup VIIb. Group VIII was divided into two subgroups: VIIIa and VIIIb (Fig 3I). The putative proteins of subgroup VIIIa had two conserved motifs, CMPeVIII-3 and -4, identified as similar to CMVIII-1 and -2 in subgroup VIIIa. The protein, PH01000069G0560, contained no motif outside the AP2/ERF domain and possessed two introns (Fig 1B), and so was assigned to subgroup VIIIb. Group IX consisted of two subgroups, IXb and IXc, with no IXa (Fig 3J). PH01000171G0880 and PH01002571G0300 genes contained two motifs, CMPeIX-4 and -5, the CMPeIX-4 motif sequence was homologous to CMIX-5 and -6 motifs; the CMPeIX-5 motif sequence was homologous to CMIX-2; the CMIX-2, -5 and -6 motifs were conserved in subgroup IXb; and so the two genes were assigned to subgroup IXb. The CMPeIX-2 and -3 motifs were conserved in PH01001229G0220, PH01000085G0010 and PH01001938G0330 genes, according to Arabidopsis and rice genes—these three genes were assigned to subgroup IXc. Group X consisted of subgroups Xa and Xb (Fig 3X). Five proteins, PH01001490G0850, PH01000493G0150, PH01000559G0630, PH01000173G0700 and PH01000437G0390, contained two conserved motifs, CMPeX-3 and -4. The CMPeX-3 motif, in the N-terminal region, was homologous to the CMX-2 motif in subgroup Xb proteins in Arabidopsis and rice, and so these five proteins were assigned to subgroup Xb. Analyses of phylogenetics and the presence of the CMPeX-2 motif indicated that PH01000004G0570, PH01001201G0350 and PH01002648G0300 genes belonged to subgroup Xa.

### Evolutionary patterns and divergence of the AP2/ERF gene family in moso bamboo and rice

**Fig 4:**
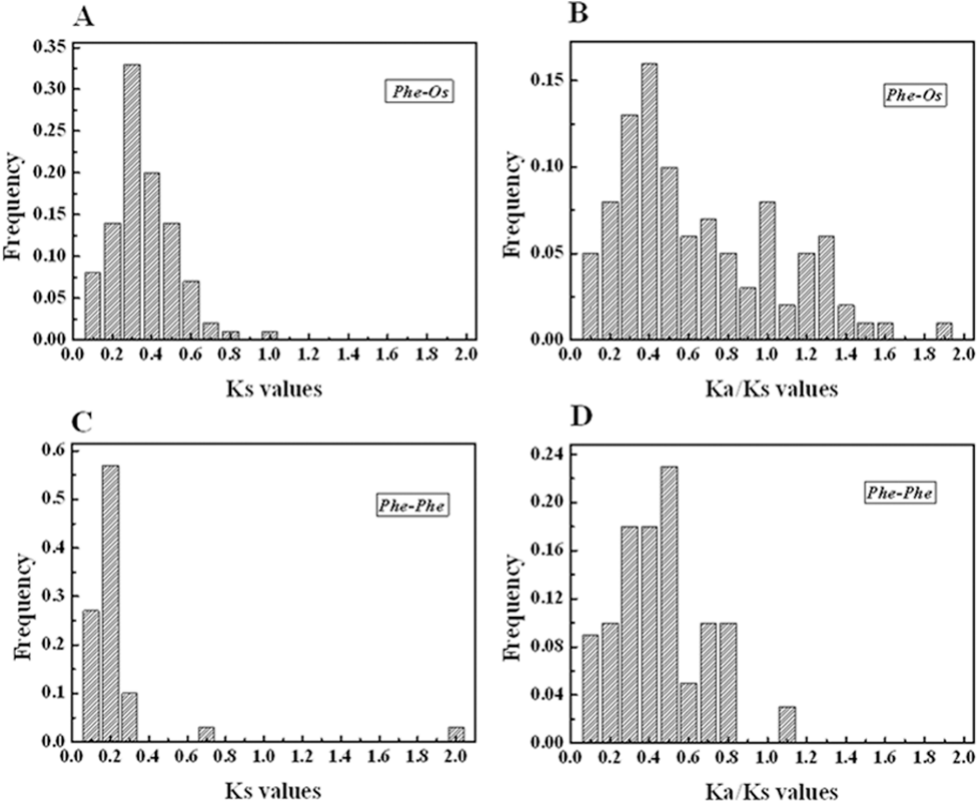
Ks and Ka/Ksvalue distributions of the AP2/ERF genes in the genomes of rice and moso bamboo viewed through the frequency distribution of relative Ks and Ka/Ks modes. Distribution of Ks and Ka/Ks values were obtained from orthologous gene-pairs (A and B) between the moso bamboo and rice genomes, and paralogous gene-pairs (C and D) in the moso bamboo genome. Ka/Ks = 1 indicates neutral selection, Ka/Ks > 1 indicates positive selection and Ka/Ks < 1 indicates purifying selection [79, 80].

In comparative genomics, the phylogeny-based and bidirectional best-hit methods are popular strategies to identify possible paralogous or orthologous genes. Using these two strategies, we found 77 putative paralogous pairs in the moso bamboo genome and 86 orthologous pairs between OsAP2/ERF and PeAP2/ERF—all gene-pairs are listed in S4 Table. In order to evaluate the divergence time between rice and moso bamboo, we used a relative Ks measure as a proxy for time—the frequency distributions of the relative Ks values obtained from duplicated orthologous and paralogous gene-pairs in the rice and moso bamboo genomes are shown in Fig 4C and 4A. The relative Ks distribution peaks around 0.2 in moso bamboo suggest a large-scale event around 15 million years ago (MYA). A recent report found that bamboo underwent whole-genome duplication 7–12 MYA [32], according to analyses of clustered gene families and gene collinearity and, when compared with the result of present study, shows that AP2/ERF family genes underwent a longer large-scale event. Similarly, the relative Ks distribution peaks at 0.2–0.3 for the duplicated orthologous gene-pairs between rice and moso bamboo indicate division within the two groups of AP2/ERF genes at 15–23 MYA. A previous study estimated that the divergence time of rice and moso bamboo was 7–15 MYA [32] and, when compared with our study, reveals that the AP2/ERF family underwent gene evolution before separation of the two progenitors. We also obtained a Ka/Ks ratio of 0.3–0.5 from the duplicated paralogous gene-pairs in the moso bamboo genome (Fig 4D); however, the duplicated orthologous gene-pairs between the rice and moso bamboo genomes gave Ka/Ks of 0.3–0.4 (Fig 4B), suggesting purifying selection for the moso bamboo genome, as well as between the rice and moso bamboo genomes.

### Analysis of the putative promoter regions of the DREB gene subfamily

Cis-regulatory elements play very important roles in determining the tissue-specific or stress-responsive expression patterns of genes [57], and multi-stimulus responsive genes are closely correlated with cis-regulatory elements in the promoter regions [58, 59]. Cis-elements located upstream of genes within 2000 bp have decisive effects on binding to target genes. To further understand transcriptional regulation and the potential functions of DREB subfamily genes in moso bamboo, 2000-bp putative promoter regions were used to identify putative stress-responsive cis-regulatory elements [60, 61]. Numerous abiotic stress cis-elements—S000176 and S000415 for drought stress, S000453 for salt stress, S00030 for heat stress, S000407 for cold stress and S000457 for wound stress—were found widely in the promoter regions of DREBs in moso bamboo, and are listed in S5 Table. This clearly showed that DREB subfamily transcription factors might respond to abiotic stress and have potential functions in enhancing abiotic stress resistance. For instance, PH01000343G0830 possessed up to 24 drought-stress elements (S000415), PH01001205G0030 had up to 34 cold-stress elements (S000407), and 24 drought-stress elements (S000415) and 34 cold-stress elements (S000407) were identified in PH01002279G0250. Further research on the function of the DREB subfamily genes in moso bamboo will be important in furthering understanding of the stress tolerance mechanism in moso bamboo.

### Expression profiles of *PeDREB*s under drought, cold and salt stresses

**Fig 5:**
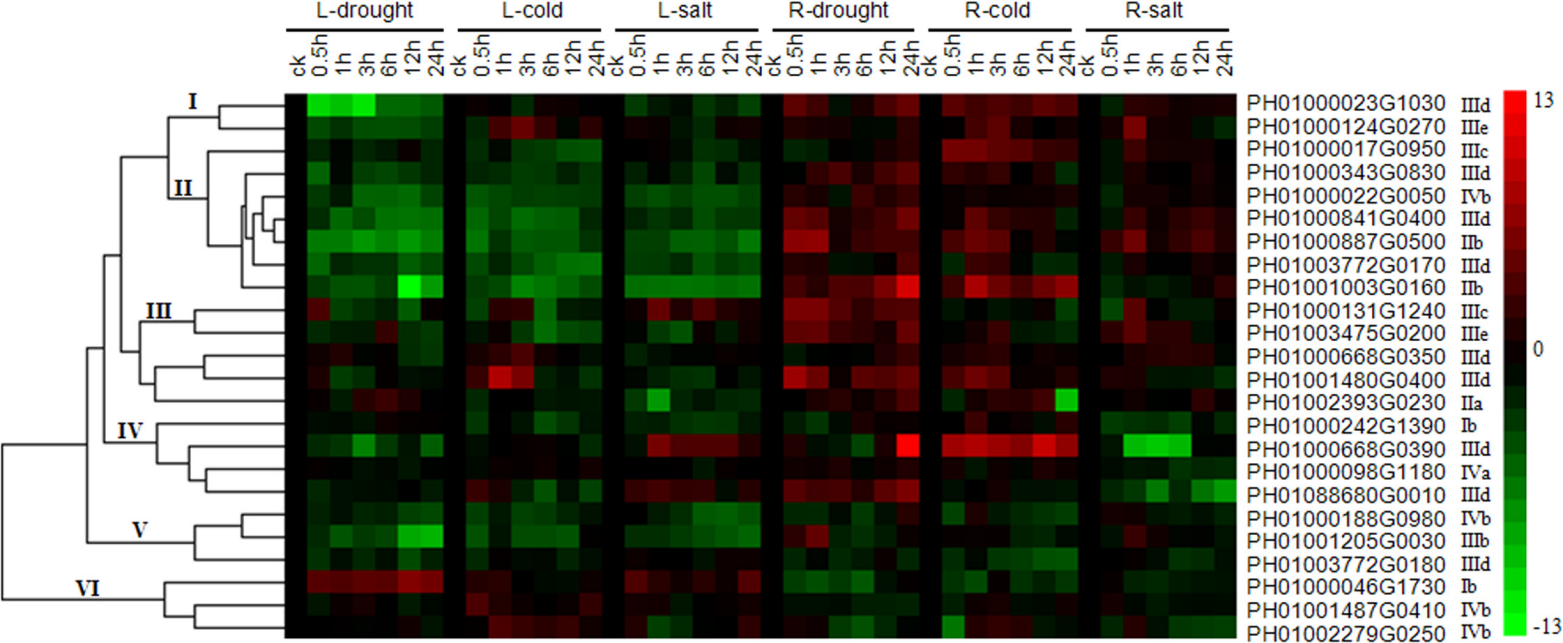
Heat map of the real-time quantitative PCR (qRT-PCR) analysis results of PeDREB genes in leaves and roots under drought, low-temperature and high salinity treatments, with three biological and technical replicates. The expression levels of genes are presented using fold-change values transformed to log_2_ format compared to controls. The log_2_ (fold-change values) and the color scale are shown at the right of the heat map.

To understand the expression profiles of the PeDREB transcription factors under drought, cold and salt stresses, 24 *PeDREB*s were analyzed by qRT-PCR. Clustering divided them into six clades: named clades I–VI (Fig 5). Clade I had two members, one belonged to IIId, the other to IIIe, and they had up-regulated expression under cold stress and down-regulated under drought and salt stresses in leaves, but were up-regulated under these three treatments in roots. Clade II had two IIb, three IIId, one IIIc and one IVb genes, and the expression levels of these seven genes showed negative responses in leaves, but strongly positive responses in roots. Clade III included one IIa, one IIIc, two IIId and one IIIe genes, the transcripts of PH01000131G1240 initially increased and then dropped at 12 h under salt treatment in leaves, and had continuous high expression levels under drought stress in roots; PH01003475G0200 had continuous high expression levels under drought stress in roots, and the expression levels under cold and salt stresses in roots initially increased and finally dropped. PH01000668G0350 and PH01001480G0400 were involved in drought and cold stress responses in leaves, they were up-regulated under the three treatments in roots; expression levels of PH01002293G0230 initially decreased and then increased under drought stress in leaves, and almost up-regulated under the three treatments in roots. Clade IV, containing one Ib, two IIId and one IVa genes, genes in clade IV were down-regulated under drought stress in leaves and salt stress in roots, had low expression levels under cold stress in leaves, was up-regulated under salt stress in leaves (excluding PH01000242G1390), and was also up-regulated under drought conditions in roots. Clade V had two IIId and one IVb genes, the expression levels of which were down-regulated or little-changed under these three stresses both in leaves and roots. Clade VI consisted of one Ib and two IVb genes, which were up-regulated in leaves and slightly down-regulated in roots under the three stresses.

## Discussion

The AP2/ERF family is one of the largest families of transcription factors, and is involved in plant development and response to abiotic and biotic stresses. Although the AP2/ERF family has been researched in various plants, this study was the first to identify and characterize the AP2/ERF family genes using the moso bamboo genome. In this study, there were fewer PeAP2/ERF genes than in other grasses, for example, 165 OsAP2/ERF [36, 37] (*Oryza sativa*), 194 ZmAP2/ERF (*Zea mays*) and 126 SvAP2/ERF [62] (*Sorghum bicolor*). A previous study showed that data from moso bamboo and rice not only reinforced the occurrence of the whole-genome duplication event but also supported a tetraploid origin of bamboo [32]. The moso bamboo genome has 24 pairs of chromosomes [63] (2n = 48), twice that of rice. Although the relationship between the two species is unclear, it is well-known that moso bamboo carries two duplicates compared to rice gene model sets [32]. It is not clear why moso bamboo contains less AP2/ERF genes than rice, and it may imply that moso bamboo suffered a recent large-scale gene loss event after the whole-genome duplication.

The structure analysis of AP2/ERF family proteins vividly revealed that the AP2 and RAV subfamilies contained two DNA binding domains; in contrast, the ERF subfamilies possessed a single AP2/ERF binding domain, showing that AP2 and RAV subfamilies members had a relatively complex structure. In moso bamboo, 52 of 116 AP2/ERF genes had introns, as for many other plants—23 of 145 AP2/ERF genes in Arabidopsis have introns, and 35 of 114 AP2/ERF family members in castor bean contain introns—it revealed that conserved intronic sites imply the evolution of introns before gene duplication. A previous study indicated the possibility that an early addition of introns or a second DNA-binding domain may have impaired the duplicative ability of the hypothesized ancestral HNH endonuclease, or a longer piece of DNA made a transposition and duplication event less likely, was consistent with the smaller number of members of AP2 and RAV subfamilies [64].

A conserved motif is a conserved amino-acid sequence with a variety of biological functions, and can be involved in transcriptional activity, protein–protein interactions and nuclear localization [7]. If proteins are classified in a subfamily or subgroup, they may share similar motifs and functions. Diverse conserved motifs within AP2/ERF family proteins have been identified in Arabidopsis and rice [7], and in this study we analyzed the motifs of each group within PeAP2/ERF family genes compared with AtAP2/ERF and OsAP2/ERF family genes. The proteins of subgroups IIb, IIIc, IIId and VIIa contain a LWSY motif at the C-terminus [65], and the LWSY motif has also been reported as conserved in A-3, A-5 and B-2 groups of the sorghum ERF family [54] and may be involved in plant cold tolerance [66]. The EAR motif has been reported to function as an active transcriptional repressor to maintain stress responses [67], for instance, the EAR-motif-containing protein Zat7 (AT3G46090) played a key role in the defense response of Arabidopsis to salinity stress [68], and RAP2.1 performed an important role in defense against cold- and drought-stresses [69]. In the present study, the EAR motif was contained in subgroups IIId, IIIc and VIIIa, indicating that these subgroup proteins may function in defense against abiotic stress. Three motifs related to putative phosphorylation sites, CMPeVI-4, CMPeVII-6 and CMPeIX-4, were conserved in subgroups VI, VIIa and IXb. CMPeVI-4/SP(T/V)SVL motif was identified in all proteins of subgroup VI in moso bamboo. The SP(T/V)SVL motif was conserved within roughly half of the CRF proteins in plants and predicted to function as a putative MAP kinase phosphorylation site. Interestingly, about half of the genes whose protein contains this motif would be shown to have altered expression through cytokinin treatment or in a cytokinin mutant background [7, 70], suggesting that the SP(T/V)SVL motif may have a important role in cytokinin response. The CMPeX-3 motif contained a characteristic conserved sequence, Cx_2_Cx_4_Cx_2~4_C, in the N-terminal region of proteins in subgroup Xb. Previous research on the Cys repeat feature, possibly a zinc-finger-like domain, suggests it has a function in response to abiotic stress. OSISAP1 is a zinc finger protein in rice, containing a Cys repeat sequence, Cx_2~4_Cx_9~12_Cx_2_Cx_4_Cx_2_Hx_5_HxC, and its over-expression in transgenic tobacco conferred tolerance to cold, dehydration and salt stress at the seed-germination/seedling stage [71]. The CMPeIII-2 motif from subgroups IIIb, IIIc and PH01000017G0950 protein is characterized by three blocks of conserved amino acid residues: LPRP, D[I/V]QAA or DIR[R/A], and LNFP. These three blocks have been identified as essential signatures in Arabidopsis for CBL-interacting serine/threonine-proteins kinase-12 [72], ethylene-responsive transcription factor ERF037 [73], dehydration responsive element binding proteins-1C and proteins-G [74], and auxin response factor-19 [75]. Regions of basic amino acid-rich sequences have often been assigned as a nuclear localization signal (NLS) in transcription factors [76, 77]. Two basic amino acid-rich sequences, KRRKRxEGKHP and KR[K/R]HS[I/K]RKRRKGKK, were characterized in CMPeIII-4 and CMPeIX-6 motifs, respectively, and may function as NLSs of proteins in groups III and IX. Liu et al. reported that regions of acid-, Gln-, Pro- and/or Ser/Thr-rich amino acid sequences were often designated as transcription activation domains [78], and several motifs identified in the present study had Ser-rich amino acid sequences, such as CMPeIII-7 and CMPeIX-6.

Investigation of gene collinearity in bamboo and rice not only supported the occurrence of the whole-genome duplication event but also the tetraploid origin of bamboo, as the most recent whole-genome duplication was likely linked to polyploidy events [32]. Recent gene duplication events play an important role in the rapid expansion and evolution of gene families, which lead to many paralogous pairs in different species [79]. Large-scale duplication events are defined as simultaneous duplications of genes. Ka and Ks are measures to explore the mechanism of gene divergence after duplication. Assuming a molecular clock, Ks of the duplicates is expected to be similar over time. There are, however, substantial rate variations among genes [80]. To better explain the patterns of macroevolution, estimates of the evolutionary rates are extremely useful [54]. To determine the relative divergence of the respective lineages, the Ks and Ka models of orthologous genes Pe–Os and paralogous genes Pe–Pe were estimated. The Ks value was calculated for each gene-pair and then used to calculate the approximate date of the duplication event (T = Ks/2λ), assuming λ of synonymous substitution of 6.5 × 10^–9^ for rice and moso bamboo [32, 53, 54]. We estimated the divergence time between rice and moso bamboo was 15–23 MYA and another large-scale event occurred approximately 15 MYA in the moso bamboo genome. Peng et al. [32] analyzed gene families and gene collinearity, and showed that bamboo underwent whole-genome duplication 7–12 MYA. A Ka/Ks value (the ratio of the rate of non-synonymous substitution to the rate of synonymous substitution) of the compared species lineage being high indicates strong selection pressure on these genes. If amino-acid replacement substitutions occurred at the same rate as synonymous substitutions, then few or no amino-acid replacement substitutions were eliminated since gene duplication, meaning Ka/Ks = 1, that is, duplicate genes had few or no selective constraints. If Ka/Ks < 1, then replacement substitutions were purged by natural selection, probably due to deleterious effects, the smaller Ka/Ks indicates greater selective constraint and the number of eliminated substitutions under which the two genes have evolved [81, 82]. The Ka/Ks value for Pe–Os was large at 0.3–0.4, implying purifying selection during their long evolutionary history. Additionally, Ka/Ks of 0.3–0.5 for Pe–Pe suggests purifying selection and strong selection constraint in moso bamboo AP2/ERF genes.

In this study, 27 DREB subfamily genes were identified and characterized, compared with 57 AtDERBs and 52 OsDREBs, indicating that there were fewer members in the DREB subfamily in moso bamboo. There were no members in each of subgroups Ia, IIc and IIIa, one member in each of subgroups IIa and IIIb, two members in each of subgroups Ib, IIb, IIIe and IVa. At present, the function of subgroup Ia genes is unknown. Two genes in Arabidopsis in subgroup Ib, *RAP2*.*4* and *RAP2*.*4B*, were highly expressed in stems and roots and were differentially induced in response to cold, dehydration and osmotic stress. However, *RAP2*.*4B* was uniquely expressed at a high level in dry seeds and was induced by heat stress, while *RAP2*.*4* was uniquely induced at a high level by salt stress [83], indicating that genes in subgroup Ib may function in different tissues and abiotic stresses. For example, *PeDREB2A* (PH01000046G1730) [84], was strongly induced by drought and salt stresses and slightly induced by cold stress in leaves. The *RAP2*.*1* (At1g46780) gene in subgroup IIa was involved in drought and cold stresses via an ABA-independent pathway in Arabidopsis [85], the *OsDERF1* (Os08g35240) gene in subgroup IIb negatively modulated ethylene synthesis and drought tolerance in rice [86] and the *SERF1* (Os05g34730) gene in subgroup IIc had root-specific induction upon salt and hydrogen peroxide stress [87]. Those genes in group II functioned in different signaling pathways and abiotic stresses. In this study, there were no subgroup IIc members; while the subgroup IIa genes were induced by drought in leaves, and strongly induced by drought and cold stresses and slightly induced by salt stress in roots; and the subgroup IIb genes were specifically highly induced by these three stresses. There is a special and thoroughly studied subgroup, IIIc, namely the DREB1s. The AtDREB1 factors are mostly induced in response to chilling [88]. Similar molecular mechanisms and physiological responses, induced and regulated by DREB1 genes, appear to occur in monocots [5, 25, 65, 89]. However, in the present study, there were only three genes in subgroup IIIc of moso bamboo, and these had multiple functions under drought, cold and salt stresses. Why moso bamboo possesses fewer DREB1 genes compared with Arabidopsis and rice is unknown. Subgroup IIId had the most *PeDREB*s, and they were mostly involved in drought, cold and salt stresses, especially in roots, but PH01003772G0180 was not induced by these stresses. The PH01000668G0350, namely *PeDREB1A*, was slightly induced by drought and cold stresses in leaves, and continuously induced by drought, cold and salt stresses in roots [84]. Some subgroup IIId genes may have constitutive expression or participate in responses to abiotic stress, for example, *OsDREB4-2* was induced by drought and salt stresses, but *OsDREB4-1* was constitutively expressed [90]. The group IV genes were designated as *DREB2* genes, although they were reported as mostly involved in drought and heat responses [88]; however, they may have more functions, such as *DREB2A* being involved in drought-, salt- and heat-stress responses in Arabidopsis [91]. *AtDREB2C*-overexpressing Arabidopsis plants were dehydration sensitive, and were freezing and heat tolerant [92]. The results of group IV factors in Arabidopsis were also accommodating in moso bamboo, function in drought, cold and salt responses. *PeDREB1* (PH01003928G0080, the expression patterns were not researched in the present study) and *PeDREB2* (PH01000098G1180) were investigated by Liu et al., and *PeDREB1* transcripts rapidly accumulated following exposure to cold stress and the expression of *PeDREB2* was induced by drought and salt stresses [93, 94]. The present study clearly revealed that the majority of *PeDREB* genes were expressed at high levels in roots under drought, cold and salt treatments; and whether they play a role in development of moso bamboo roots will require further investigation.

## Conclusion

In the current study, we identified 116 AP2/ERF family factors in the moso bamboo genome and characterized the *PeDREB* genes’ expression profiles under drought, low-temperate and salinity conditions. A comparison of homologs from genomes of other species, together with their expression patterns, may help in understanding of the role of these proteins in plants. Moso bamboo represents the only Bambusoideae plant whose genome has been sequenced and whose economic, ecological and social values are very high. Surveying the role of *PeDREB* genes under abiotic stress in this species may help to overcome challenges in cultivating moso bamboo.

## Supporting Information

S1 Fig Comparison of amino acid sequences of the AP2/ERF domains of the 116 PeAP2/ERF proteins. The AP2/ERF domain sequences were aligned using ClustalW 1.83 software. The colored background represents the conserved amino acid residues (> 85%). The red star symbol represents the AP2/ERF domain sequence of groups VI–L proteins, the triangle symbol represents the AP2/ERF domain sequence of the soloist protein, and the short line symbol represents the AP2/ERF domain sequence of the AP2 subfamily proteins that possess a single domain.
(TIF)

S2 Fig Unrooted phylogenetic tree of the AP2/ERF domain sequences in Arabidopsis, rice and moso bamboo. The AP2/ERF domain sequences of 135 AtAP2/ERF, 147 OsAP2/ERF and 116 PeAP2/ERF proteins were aligned using ClustalW 1.83 software, and the phylogenetic tree was generated using the NJ method. The names of every subfamily or group were reported by Nakano et al. (2006) [7].
(TIF)

S3 Fig Gene motifs within the AP2 subfamily and the RAV subfamily or soloist genes in moso bamboo, Arabidopsis and rice. The clades of the phylogenetic tree detected within moso bamboo, Arabidopsis and rice AP2, RAV or soloist genes. The conserved motifs were identified in the proteins of every subfamily, AP2 subfamily (A), RAV subfamily (B) and soloist (C). Each colored box below the tree represents the conserved motifs. The CMPeR-1 and CMPeS-1 motifs represent the AP2/ERF domain in AP2 and soloist proteins.
(TIF)

S1 Table Forward and reverse primers used in qRT-PCR gene expression studies. (DOC)

S2 Table Putative 121 AP2/ERF family genes identified in moso bamboo. (XLS)

S3 Table Motif sequences of AP2/ERF genes identified in moso bamboo using MEME tools. (XLS)

S4 Table Summary of the duplicated gene-pairs and determination of Ka and Ks values of the AP2/ERF family transcription factors in moso bamboo and rice. Blank cells indicate that the Ka and Ks values were not determined.
(XLS)

S5 Table Summary of abiotic stress inducible cis-elements in the promoter regions of DREB subfamily genes in moso bamboo. *Cis*-elements with larger numbers are marked in red.
(DOC)
