# Supplementary figures and images for: Genome-Wide Analysis of the AP2/ERF Transcription Factors Family and the Expression Patterns of DREB Genes in Moso Bamboo (Phyllostachys edulis)
Source: PLoS One. 2015 May 18;10(5):e0126657. doi: 10.1371/journal.pone.0126657 (PMC4436012; doi:10.1371/journal.pone.0126657)

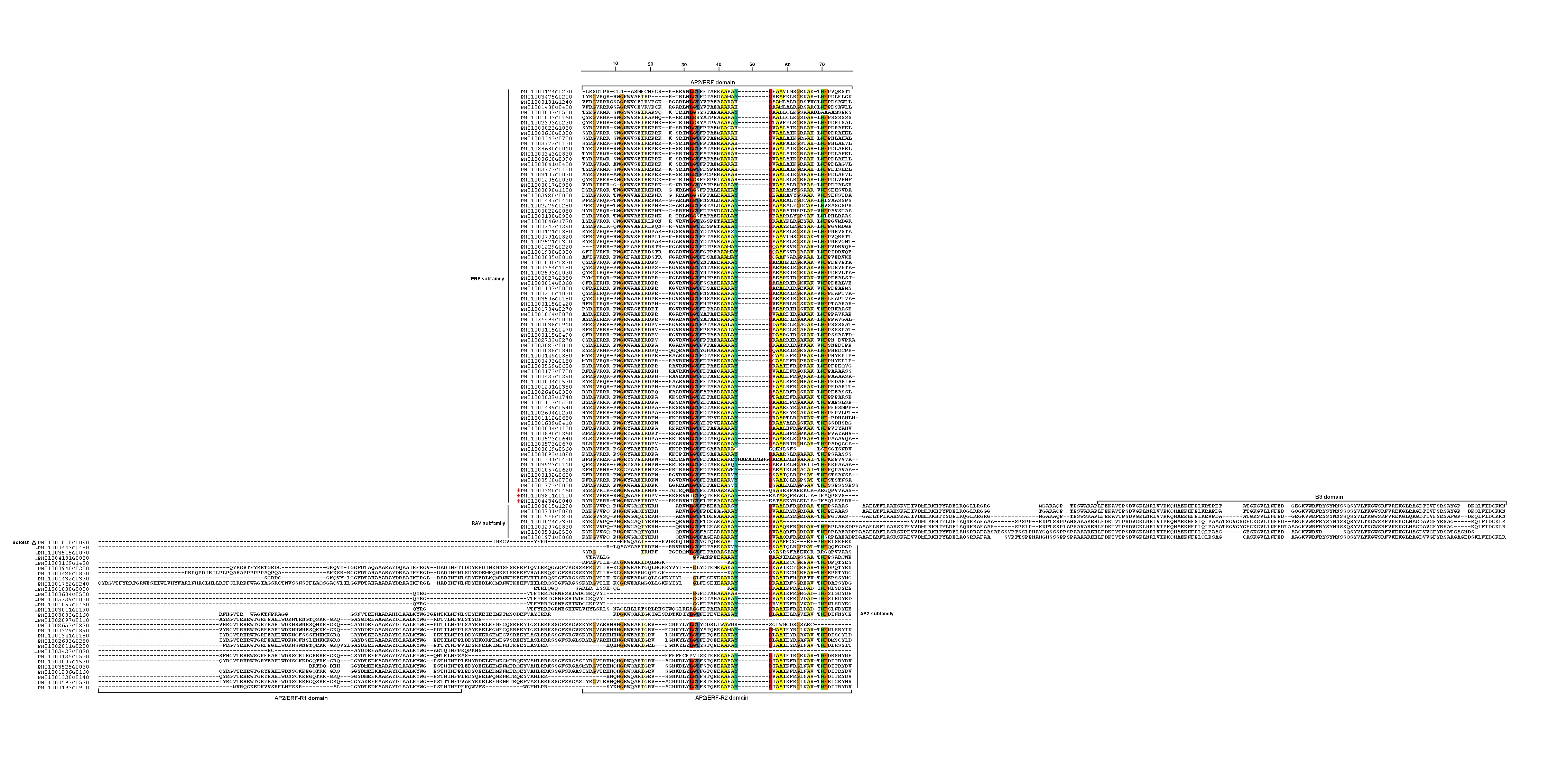

Supplement: S1 Fig — The AP2/ERF domain sequences were aligned using ClustalW 1.83 software. The colored background represents the conserved amino acid residues (> 85%). The red star symbol represents the AP2/ERF domain sequence of groups VI–L proteins, the triangle symbol represents the AP2/ERF domain sequence of the soloist protein, and the short line symbol represents the AP2/ERF domain sequence of the AP2 subfamily proteins that possess a single domain. (TIF) [file pone.0126657.s001.tif]

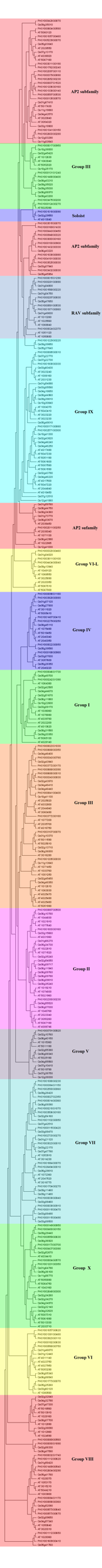

Supplement: S2 Fig — The AP2/ERF domain sequences of 135 AtAP2/ERF, 147 OsAP2/ERF and 116 PeAP2/ERF proteins were aligned using ClustalW 1.83 software, and the phylogenetic tree was generated using the NJ method. The names of every subfamily or group were reported by Nakano et al. (2006) [7]. (TIF) [file pone.0126657.s002.tif]

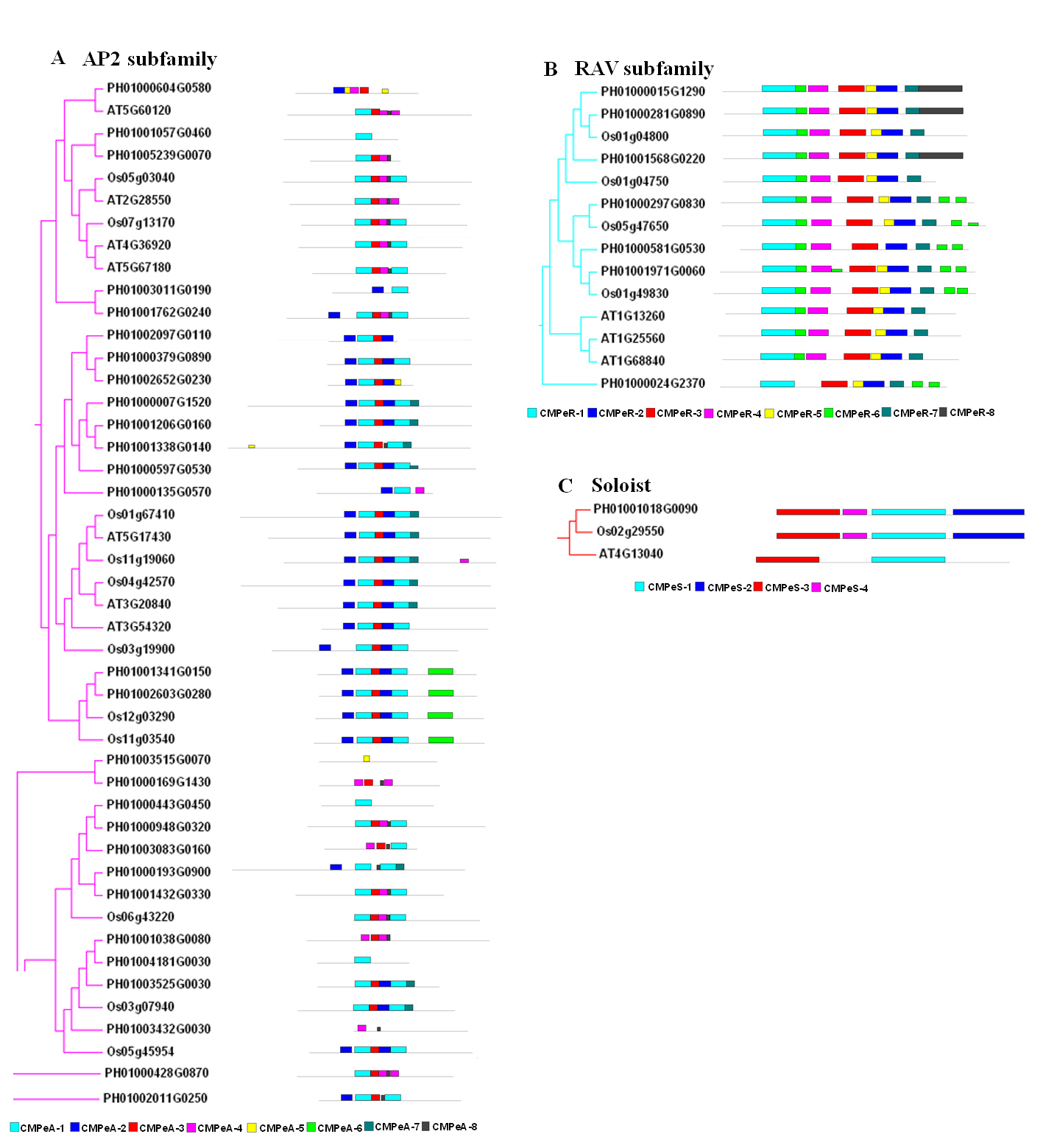

Supplement: S3 Fig — The clades of the phylogenetic tree detected within moso bamboo, Arabidopsis and rice AP2, RAV or soloist genes. The conserved motifs were identified in the proteins of every subfamily, AP2 subfamily (A), RAV subfamily (B) and soloist (C). Each colored box below the tree represents the conserved motifs. The CMPeR-1 and CMPeS-1 motifs represent the AP2/ERF domain in AP2 and soloist proteins. (TIF) [file pone.0126657.s003.tif]
