# Supplementary material for: Genome-Wide Analysis of the AP2/ERF Transcription Factors Family and the Expression Patterns of DREB Genes in Moso Bamboo (Phyllostachys edulis)
Source: PLoS One. 2015 May 18;10(5):e0126657. doi: 10.1371/journal.pone.0126657 (PMC4436012; doi:10.1371/journal.pone.0126657)
Supplement: S1 Table — (DOC) [file pone.0126657.s004.doc]

**Table S1 Forward and reverse primers used in the qRT-PCR genes expression studies**

| Gene name | Sequence(5’to 3’) | Gene name | Sequence(5’to 3’) |
| --- | --- | --- | --- |
| PH01000017G0950-F | CGCTAAACTTCCCCGACAC | PH01000668G0390-F | CCGTCACCTCCTCTTCGT |
| PH01000017G0950-R | AAAACGTCATCCTCGTCCAC | PH01000668G0390-R | CTCCTGGACATCCTTGGG |
| PH01000022G0050-F | CGGAGAACATGAAACACA | PH01000791G0820 -F | CTACGAGCAACCTGTCCC |
| PH01000022G0050-R | GGAAAGAGAACAAGACGC | PH01000791G0820 -R | TCCTCTCCTCATCATCCA |
| PH01000023G1030-F | TCGTCCTTGTCCATTTCC | PH01000841G0400-F | TCCTCCACCGTCACGGAC |
| PH01000023G1030-R | TGCTGCTCTCCTCTCCCT | PH01000841G0400-R | CGACACCCACTTGCCCCA |
| PH01000046G1730-F | CGAGGAGAACAAGGAGAACG | PH01000887G0500-F | TCGTGGGTGTCGGAGATC |
| PH01000046G1730-R | ATCAACTCGGGATCAAACGA | PH01000887G0500-R | GCTGCAGGCCGGGGTGGT |
| PH01000098G1180-F | AGAATGCCTCAAGGAAAGCA | PH01001003G0160-F | GATAATCGCCACTCGGACTC |
| PH01000098G1180-R | GTGCAGCTTCCAATGCAGTA | PH01001003G0160-R | CGGCGATAAAATCCATGAAC |
| PH01000131G1240-F | CACCGTGTGCCTCAACTTC | PH01001205G0030-F | CGGCACAGGTCCGGTTTA |
| PH01000131G1240-R | TTTCCCCGAACAAGTCAAAC | PH01001205G0030-R | AGACGGGTCCCAGAGCAA |
| PH01000188G0980-F | ACGTCTCCTTGCTCCACTGT | PH01001480G0400-F | GGGGAAATGGACTTGGGTAT |
| PH01000188G0980-R | CTTGAGCACGCCTATCTGCT | PH01001480G0400-R | TAGCTCCAGAGTGCGACATC |
| PH01000242G1390-F | AGCAAGGAGAATCCATGCAC | PH01001487G0410-F | CGTCAGGACAAGCCCTCA |
| PH01000242G1390-R | ATCAACTCGGGATCAAACGA | PH01001487G0410-R | AACACCTCGAGGGGCAC |
| PH01000343G0830-F | CCGACCTACGCCAATGCT | PH01002279G0250-F | TGCAGCCTCGTCCTCCAC |
| PH01000343G0830-R | ACGGAGGGCCGTATCTGA | PH01002279G0250-R | CCTCCTCTCTTGTCATCT |
| PH01000124G0270-F | GTCGGCGTCTGTTTCTTCTT | PH01002393G0230-F | AGATCTCAGCGCTGACCCT |
| PH01000124G0270-R | TAATCTCCTGCAACCCATCC | PH01002393G0230-R | TGGCCTTCTTCCGGATCGA |
| PH01000668G0350-F | CCTCCTCGTTGTTGTCCACT | PH01003475G0200-F | GAACCCGAACAAAATCCTCA |
| PH01000668G0350-R | GCTCTCCTCTCCCTGTCCAT | PH01003475G0200-R | GTAGTCCAATCCGGTGTGCT |
| PH01003772G0170-F | GCCGCGTTCGCCATCAAG | PH01088680G0010-F | CACCGAAAGATGTCCAGG |
| PH01003772G0170-R | CACCACAGCCCGTCCCTC | PH01088680G0010-R | AGTATTCCCACAGCAGCG |
| PH01003772G0180-F | CGCCGGTGTCCAATGGTT | TIP41-F | AAAATCATTGTAGGCCATTGTCG |
| PH01003772G0180-R | CGGTACGTCGGGTGCTTC | TIP41-R | ACTAAATTAAGCCAGCGGGAGTG |
