# Supplementary material for: Genome-Wide Analysis of the AP2/ERF Transcription Factors Family and the Expression Patterns of DREB Genes in Moso Bamboo (Phyllostachys edulis)
Source: PLoS One. 2015 May 18;10(5):e0126657. doi: 10.1371/journal.pone.0126657 (PMC4436012; doi:10.1371/journal.pone.0126657)
Supplement: S5 Table — Cis-elements with larger numbers are marked in red. (DOC) [file pone.0126657.s008.doc]

**Table S5 Summary of abiotic-stress inducible cis-elements is in the promoter regions of DREB subfamily genes in moso bamboo.** *Cis*-elements with larger numbers were marked red. Motif S000415 ACGT is overlapped with motif S000414 ACGTG and S000133 CCACGTGG, and motif S000153 CCGAC is overlapped with motif S000402 ACCGAC and S000418 RCCGAC, so total number of motifs was based on specific position of motifs located on the promoter.

| **Abiotic**  **stress** | **Gene name**  ***cis*-element** | **motif sequence** | ***PH01000017G0950*** | ***PH01000022G0050*** | ***PH01000023G1030*** | ***PH01000046G1730*** | ***PH01000098G1180*** | ***PH01000131G1240*** | ***PH01000188G0980*** |
| --- | --- | --- | --- | --- | --- | --- | --- | --- | --- |
| Drought-stress | **S000133** | **CCACGTGG** | **0** | **0** | **0** | **0** | **0** | **0** | **0** |
|  | S000153 | CCGAC | 1 | 3 | 1 | 2 | 2 | 3 | 2 |
|  | S000174 | CACATG | 2 | 0 | 2 | 3 | 2 | 1 | 2 |
|  | S000175 | CTAACCA | 0 | 0 | 0 | 0 | 0 | 0 | 0 |
|  | S000176 | CNGTTR | 4 | 3 | 5 | 3 | 3 | 4 | 4 |
|  | S000177 | TAACTG | 0 | 0 | 0 | 0 | 0 | 1 | 0 |
|  | **S000402** | **ACCGAC** | **0** | **1** | **0** | **1** | **0** | **0** | **2** |
|  | S000408 | WAACCA | 2 | 2 | 2 | 4 | 2 | 4 | 2 |
|  | S000413 | CATGTG | 2 | 0 | 0 | 3 | 2 | 1 | 2 |
|  | **S000414** | **ACGTG** | **3** | **1** | **3** | **0** | **2** | **2** | **1** |
|  | S000415 | ACGT | 6 | 11 | 14 | 10 | 6 | 10 | 8 |
|  | **S000418** | **RCCGAC** | **0** | **1** | **1** | **2** | **0** | **1** | **2** |
|  | total |  | 17 | 20 | 25 | 25 | 17 | 24 | 20 |
| Cold-  stress | S000153 | CCGAC | 1 | 3 | 1 | 2 | 2 | 3 | 2 |
|  | **S000402** | **ACCGAC** | **0** | **1** | **0** | **1** | **0** | **0** | **2** |
|  | S000407 | CANNTG | 14 | 14 | 18 | 27 | 20 | 4 | 24 |
|  | **S000418** | **RCCGAC** | **0** | **1** | **1** | **2** | **0** | **1** | **2** |
|  | total |  | 15 | 17 | 19 | 29 | 22 | 7 | 26 |
| Salt-  stress | S000402 | ACCGAC | 0 | 1 | 0 | 1 | 0 | 0 | 2 |
|  | S000418 | RCCGAC | 0 | 1 | 1 | 2 | 0 | 1 | 2 |
|  | S000453 | GAAAAA | 2 | 0 | 0 | 3 | 1 | 0 | 0 |
|  | total |  | 2 | 2 | 1 | 6 | 1 | 1 | 4 |
| Heat-  stress | S000030 | CCAAT | 3 | 4 | 7 | 3 | 2 | 7 | 7 |
|  | S000418 | RCCGAC | 0 | 1 | 1 | 2 | 0 | 1 | 2 |
|  | total |  | 3 | 5 | 8 | 5 | 2 | 8 | 9 |
| Wound-stress | S000244 | AACGTGT | 1 | 0 | 0 | 1 | 0 | 0 | 0 |
|  | S000444 | AGATCCAA | 0 | 0 | 0 | 0 | 0 | 0 | 0 |
|  | S000457 | TGACY | 3 | 5 | 4 | 3 | 3 | 7 | 2 |
|  | total |  | 4 | 5 | 4 | 4 | 3 | 7 | 2 |

| **Abiotic**  **stress** | **Gene name**  ***cis*-element** | **motif Sequence** | PH01000242G1390 | ***PH01000343G0780*** | ***PH01000124G0270*** | ***PH01000343G0830*** | PH01000668G0350 | ***PH01000668G0390*** |
| --- | --- | --- | --- | --- | --- | --- | --- | --- |
| Drought-stress | **S000133** | **CCACGTGG** | **0** | **0** | **0** | **0** | **0** | **0** |
|  | S000153 | CCGAC | 1 | 0 | 0 | 1 | 4 | 3 |
|  | S000174 | CACATG | 3 | 1 | 2 | 0 | 3 | 1 |
|  | S000175 | CTAACCA | 1 | 0 | 0 | 0 | 0 | 0 |
|  | S000176 | CNGTTR | 10 | 6 | 4 | 7 | 9 | 5 |
|  | S000177 | TAACTG | 0 | 1 | 1 | 0 | 0 | 0 |
|  | **S000402** | **ACCGAC** | **1** | **0** | **0** | **0** | **2** | **0** |
|  | S000408 | WAACCA | 2 | 1 | 2 | 4 | 6 | 2 |
|  | S000413 | CATGTG | 3 | 1 | 2 | 0 | 3 | 1 |
|  | **S000414** | **ACGTG** | **1** | **1** | **1** | **6** | **3** | **5** |
|  | S000415 | ACGT | 6 | 2 | 10 | 24 | 8 | 16 |
|  | **S000418** | **RCCGAC** | **1** | **0** | **0** | **1** | **2** | **2** |
|  | total |  | 26 | 12 | 21 | 35 | 33 | 28 |
| Cold-  stress | S000153 | CCGAC | 1 | 0 | 0 | 1 | 4 | 3 |
|  | **S000402** | **ACCGAC** | **1** | **0** | **0** | **0** | **2** | **0** |
|  | S000407 | CANNTG | 24 | 19 | 24 | 14 | 28 | 20 |
|  | **S000418** | **RCCGAC** | **1** | **0** | **0** | **1** | **2** | **2** |
|  | total |  | 25 | 19 | 24 | 15 | 32 | 23 |
| Salt-  stress | S000402 | ACCGAC | 1 | 0 | 0 | 0 | 2 | 0 |
|  | S000418 | RCCGAC | 1 | 0 | 0 | 1 | 2 | 2 |
|  | S000453 | GAAAAA | 5 | 0 | 0 | 0 | 5 | 2 |
|  | total |  | 7 | 0 | 0 | 1 | 9 | 4 |
| Heat-  stress | S000030 | CCAAT | 4 | 4 | 6 | 5 | 9 | 7 |
|  | S000418 | RCCGAC | 1 | 0 | 0 | 1 | 2 | 2 |
|  | total |  | 5 | 4 | 6 | 6 | 11 | 9 |
| Wound-stress | S000244 | AACGTGT | 0 | 0 | 0 | 0 | 0 | 0 |
|  | S000444 | AGATCCAA | 0 | 0 | 0 | 0 | 0 | 0 |
|  | S000457 | TGACY | 5 | 8 | 10 | 3 | 8 | 3 |
|  | total |  | 5 | 8 | 10 | 3 | 8 | 3 |

| **Abiotic**  **stress** | **Gene name**  ***cis*-element** | **motif sequence** | ***PH01000841G0400*** | ***PH01000887G0500*** | | ***PH01001003G0160*** | | ***PH01001205G0030*** | | ***PH01001480G0400*** | | ***PH01001487G0410*** | ***PH01002279G0250*** | |
| --- | --- | --- | --- | --- | --- | --- | --- | --- | --- | --- | --- | --- | --- | --- |
| Drought-stress | **S000133** | **CCACGTGG** | **0** | **0** | | **0** | | **0** | | **0** | | **0** | **2** | |
|  | S000153 | CCGAC | 1 | 1 | | 0 | | 3 | | 1 | | 4 | 2 | |
|  | S000174 | CACATG | 3 | 0 | | 1 | | 6 | | 3 | | 1 | 2 | |
|  | S000175 | CTAACCA | 0 | 0 | | 0 | | 0 | | 0 | | 0 | 0 | |
|  | S000176 | CNGTTR | 5 | 0 | | 9 | | 4 | | 6 | | 3 | 4 | |
|  | S000177 | TAACTG | 0 | 0 | | 0 | | 0 | | 0 | | 0 | 0 | |
|  | **S000402** | **ACCGAC** | **0** | **0** | | **0** | | **1** | | **0** | | **1** | **1** | |
|  | S000408 | WAACCA | 1 | 0 | | 4 | | 3 | | 2 | | 2 | 3 | |
|  | S000413 | CATGTG | 3 | 0 | | 1 | | 6 | | 3 | | 1 | 2 | |
|  | **S000414** | **ACGTG** | **2** | **0** | | **0** | | **2** | | **6** | | **1** | **8** | |
|  | S000415 | ACGT | 4 | 0 | | 0 | | 8 | | 18 | | 2 | 24 | |
|  | **S000418** | **RCCGAC** | **0** | **0** | | **0** | | **1** | | **0** | | **2** | **2** | |
|  | total |  | 17 | 1 | | 15 | | 30 | | 33 | | 13 | 27 | |
| Cold-  stress | S000153 | CCGAC | 0 | 1 | | 0 | | 3 | | 1 | | 4 | 2 | |
|  | **S000402** | **ACCGAC** | **0** | **0** | | **0** | | **1** | | **0** | | **1** | **1** | |
|  | S000407 | CANNTG | 28 | 4 | | 26 | | 34 | | 30 | | 14 | 34 | |
|  | **S000418** | **RCCGAC** | **0** | **0** | | **0** | | **1** | | **0** | | **2** | **2** | |
|  | total |  | 28 | 5 | | 26 | | 37 | | 31 | | 18 | 36 | |
| Salt-  stress | S000402 | ACCGAC | 0 | 0 | | 0 | | 1 | | 0 | | 1 | 1 | |
|  | S000418 | RCCGAC | 0 | 0 | | 0 | | 1 | | 0 | | 2 | 2 | |
|  | S000453 | GAAAAA | 5 | 0 | | 1 | | 0 | | 0 | | 0 | 3 | |
|  | total |  | 5 | 0 | | 1 | | 2 | | 0 | | 3 | 6 | |
| Heat-  stress | S000030 | CCAAT | 4 | 1 | | 7 | | 3 | | 2 | | 1 | 3 | |
|  | S000418 | RCCGAC | 0 | 0 | | 0 | | 1 | | 0 | | 2 | 2 | |
|  | total |  | 4 | 1 | | 7 | | 4 | | 2 | | 3 | 5 | |
| Wound-stress | S000244 | AACGTGT | 1 | 0 | | 0 | | 0 | | 0 | | 0 | 0 | |
|  | S000444 | AGATCCAA | 0 | 0 | | 0 | | 0 | | 0 | | 0 | 0 | |
|  | S000457 | TGACY | 7 | 2 | | 7 | | 9 | | 10 | | 4 | 4 | |
|  | total |  | 8 | 2 | 7 | | 9 | | 10 | | 4 | | 4 |  |

| **Abiotic**  **stress** | **Gene name**  **cis-element** | **motif sequence** | ***PH01002393G0230*** | ***PH01003107G0070*** | ***PH01003475G0200*** | ***PH01003772G0170*** | ***PH01003772G0180*** | ***PH01003928G0080*** | ***PH01088680G0010*** |
| --- | --- | --- | --- | --- | --- | --- | --- | --- | --- |
| Drought-stress | **S000133** | **CCACGTGG** | **0** | **0** | **0** | **0** | **0** | **0** | **0** |
|  | S000153 | CCGAC | 2 | 4 | 4 | 4 | 2 | 6 | 0 |
|  | S000174 | CACATG | 3 | 2 | 1 | 2 | 1 | 0 | 0 |
|  | S000175 | CTAACCA | 0 | 1 | 0 | 1 | 0 | 0 | 0 |
|  | S000176 | CNGTTR | 11 | 6 | 3 | 5 | 8 | 4 | 0 |
|  | S000177 | TAACTG | 0 | 0 | 0 | 0 | 1 | 0 | 0 |
|  | **S000402** | **ACCGAC** | **1** | **2** | **1** | **1** | **0** | **1** | **0** |
|  | S000408 | WAACCA | 1 | 0 | 5 | 6 | 1 | 0 | 0 |
|  | S000413 | CATGTG | 3 | 2 | 1 | 2 | 1 | 0 | 0 |
|  | **S000414** | **ACGTG** | **3** | **0** | **2** | **0** | **8** | **0** | **0** |
|  | S000415 | ACGT | 10 | 4 | 8 | 6 | 14 | 0 | 0 |
|  | **S000418** | **RCCGAC** | **2** | **2** | **1** | **3** | **0** | **3** | **0** |
|  | total |  | 30 | 19 | 22 | 26 | 28 | 11 | 0 |
| Cold-  stress | S000153 | CCGAC | 2 | 4 | 4 | 4 | 2 | 6 | 0 |
|  | **S000402** | **ACCGAC** | **1** | **2** | **1** | **1** | **0** | **1** | **0** |
|  | S000407 | CANNTG | 26 | 27 | 14 | 18 | 16 | 6 | 0 |
|  | **S000418** | **RCCGAC** | **2** | **2** | **1** | **3** | **0** | **3** | **0** |
|  | total |  | 28 | 31 | 18 | 22 | 18 | 12 | 0 |
| Salt-  stress | S000402 | ACCGAC | 2 | 2 | 1 | 1 | 0 | 1 | 0 |
|  | S000418 | RCCGAC | 2 | 2 | 1 | 3 | 0 | 3 | 0 |
|  | S000453 | GAAAAA | 3 | 0 | 3 | 7 | 0 | 1 | 0 |
|  | total |  | 7 | 4 | 5 | 11 | 0 | 5 | 0 |
| Heat-  stress | S000030 | CCAAT | 6 | 6 | 6 | 3 | 5 | 4 | 0 |
|  | S000418 | RCCGAC | 2 | 2 | 1 | 3 | 0 | 3 | 0 |
|  | total |  | 8 | 8 | 7 | 6 | 5 | 7 | 0 |
| Wound-stress | S000244 | AACGTGT | 0 | 0 | 1 | 0 | 0 | 0 | 0 |
|  | S000444 | AGATCCAA | 2 | 0 | 0 | 0 | 2 | 0 | 0 |
|  | S000457 | TGACY | 8 | 5 | 5 | 4 | 10 | 3 | 0 |
|  | total |  | 10 | 5 | 6 | 4 | 12 | 3 | 0 |
